# Tissue microRNA Profiling Identifies Prognostic Signatures in Prostate Cancer and Highlights CPEB3 as a Candidate Biomarker

**DOI:** 10.3390/biomedicines14051169

**Published:** 2026-05-21

**Authors:** Jae-Heon Kim, Ah-Rim Moon, Miho Song, Kwang-Woo Lee, Soo Min Suh, Hui Ji Kim, Luis Alfonso Pefianco, Kevin Andrean, Seongho Ryu, Yun-Seob Song

**Affiliations:** 1 Department of Urology, Soonchunhyang University School of Medicine, Seoul 04404, Republic of Korea; piacekjh@hanmail.net (J.-H.K.); miho@schmc.ac.kr (M.S.); 2 Department of Pathology, Soonchunhyang University School of Medicine, Bucheon 14584, Republic of Korea; armoon@schmc.ac.kr; 3 Department of Urology, Soonchunhyang University School of Medicine, Bucheon 14584, Republic of Korea; urolkw@schmc.ac.kr; 4 Soonchunhyang Institute of Medi-Bio Science (SIMS), Soonchunhyang University, Cheonan 31151, Republic of Korea; niceolvia@naver.com (S.M.S.); khjee1932@gene2us.com (H.J.K.); luispefianco@sch.ac.kr (L.A.P.); kevinandrean@sch.ac.kr (K.A.); 5 Department of Integrated Biomedical Science, Soonchunhyang University, Cheonan 31151, Republic of Korea

**Keywords:** prostate cancer, microRNA, next-generation sequencing, biochemical recurrence, CPEB3

## Abstract

**Purpose**: Prostate cancer is one of the most common malignancies in men, yet current prognostic methods remain suboptimal. Emerging evidence indicates that microRNAs (miRNAs) play critical roles in prostate cancer progression. This study aimed to identify miRNAs associated with adverse clinical outcomes by comparing miRNA expression profiles between prostate tumors with unfavorable versus favorable prognostic features. **Materials and Methods**: High-throughput next-generation sequencing (NGS) was used to analyze miRNA expression in formalin-fixed, paraffin-embedded prostate cancer tissue samples. Patients were classified into favorable or unfavorable prognosis groups based on risk stratification scores, Gleason grade group, and biochemical recurrence. Differentially expressed miRNAs were identified using a fold-change threshold ≥2 and a false discovery rate (FDR) <0.05. Predicted target genes and pathway analyses were conducted to generate candidate regulatory hypotheses rather than confirm mechanistic relationships. **Results**: Several miRNAs were differentially expressed according to prognostic category. miR-206 was significantly downregulated in high-risk tumors compared with low-risk tumors. High-Gleason-grade tumors showed reduced expression of miR-7704 and miR-4454, while miR-25-3p and let-7f-5p were upregulated. In patients with early biochemical recurrence, miR-7704 and miR-10400-5p were downregulated relative to those with prolonged recurrence-free survival. Target prediction analysis identified CPEB3, HAND1, PTAR1, and SPRYD4 as shared candidate targets, with CPEB3 emerging as a prioritized candidate supported by consistency in external datasets rather than a confirmed molecular target. **Conclusions:** Distinct miRNA expression patterns correlate with prostate cancer aggressiveness and clinical outcomes. miR-206, miR-7704, miR-4454, miR-25-3p, and let-7f-5p represent candidate prognostic biomarkers. Their shared target CPEB3 should be interpreted as a prioritized candidate for future investigation. Given the very small sample size and the lack of qRT-PCR and functional validation, these findings should be considered preliminary and hypothesis-generating, requiring validation in larger independent cohorts and experimental studies.

## 1. Introduction

Prostate cancer is one of the most frequently diagnosed malignancies and remains a leading cause of cancer-related mortality among men worldwide [1]. Although patients with localized disease generally experience favorable long-term outcomes [2,3], prognosis declines sharply once the cancer advances or metastasizes [4,5]. This marked contrast has prompted intensive investigation into the molecular mechanisms driving prostate cancer progression, with the goal of identifying new therapeutic targets. Among the molecular factors under study, microRNAs (miRNAs) have emerged as key regulators in cancer biology.

miRNAs are small (approximately 18–24 nucleotides), non-coding RNAs that modulate gene expression at the post-transcriptional level by inhibiting mRNA translation or promoting mRNA degradation [6,7]. They are involved in a wide range of biological processes, including cell growth, differentiation, apoptosis, metabolism, metastasis, and immune regulation [8]. Consequently, aberrant miRNA expression can disrupt critical cellular pathways and contribute to cancer initiation and progression.

Prostate cancer exhibits pronounced molecular heterogeneity [9], highlighting the need for disease- and stage-specific biomarkers to enhance diagnostic accuracy, prognostic assessment, and therapeutic decision-making. In addition to conventional protein markers and mRNAs, miRNAs have gained considerable attention as potential biomarkers. Increasing evidence suggests that prostate- or cancer-specific miRNA signatures can distinguish malignant from benign prostate conditions, such as benign prostatic hyperplasia, and reflect tumor aggressiveness [10,11]. It is estimated that miRNAs regulate up to 30% of human protein-coding genes, and numerous miRNAs are dysregulated in prostate cancer compared with normal prostate tissue [12]. These alterations can reshape gene regulatory networks and signaling pathways that promote tumor development and progression [13,14]. Dysregulated miRNAs may facilitate oncogenesis by suppressing tumor-suppressor genes or by disrupting miRNAs that normally restrain tumor growth, thereby influencing proliferation, differentiation, metabolic reprogramming, apoptosis, senescence, and immune evasion [10,14,15].

Importantly, miRNAs are stable and readily detectable in biological fluids, including blood and urine [16], and advances in detection technologies now allow robust miRNA profiling from clinical specimens [17]. These characteristics make miRNAs attractive candidates for minimally invasive cancer biomarkers.

Despite growing interest, relatively few studies have investigated tissue-based miRNA expression in prostate cancer in relation to clinically relevant prognostic outcomes. Most prior studies have focused on circulating miRNAs or on distinguishing malignant from benign tissue, rather than stratifying tumors according to disease aggressiveness or recurrence risk. In addition, many existing studies have been limited by heterogeneity in study design and lack of integration across multiple prognostic dimensions.

Given these gaps, and considering the limited sample size available in the present study, our objective was not to establish definitive biomarkers or therapeutic targets, but rather to perform an exploratory, hypothesis-generating analysis. Using NGS-based miRNA profiling, we compared tumors with favorable and unfavorable prognostic features across multiple clinically relevant stratifications. Furthermore, we conducted target prediction and pathway analyses to identify candidate regulatory networks and potential downstream targets for future investigation. Accordingly, the findings of this study should be interpreted as preliminary and intended to generate biologically plausible hypotheses that warrant validation in larger, independent cohorts and functional studies.

## 2. Materials and Methods

### 2.1. Patients and Sample Collection

This retrospective study included 18 patients diagnosed with prostatic adenocarcinoma who underwent surgical resection at Soonchunhyang University Hospital between January 2002 and December 2012. The diagnosis of prostate adenocarcinoma was confirmed by histopathological examination, including Gleason grading and immunohistochemical analysis. Tumor specimens were processed as formalin-fixed, paraffin-embedded (FFPE) tissues following standard clinical protocols. Representative tumor regions with sufficient tumor cellularity were selected by experienced pathologists based on hematoxylin and eosin (H&E)-stained slides, and the corresponding areas were microdissected from FFPE blocks for RNA extraction. Clinical and pathological information, including Gleason score/grade, pathological stage, prostate-specific antigen (PSA) levels, and follow-up data, was retrieved from medical records. According to the policy of the Ethics Committee of Institutional Review Boards of Soonchunhyang University Seoul Hospital (No. 2017-02-002) and Bucheon Hospital (No. 2017-03-004), informed consent for publication was not required for this study. This exemption is in accordance with the institutional policy of Institutional Review Boards of Soonchunhyang University Seoul Hospital and Bucheon Hospital, which states that prior ethics approval is not required.

Patients were stratified into prognostic subgroups based on three criteria: (i) clinical risk category (low risk vs. high risk; n = 3 per group), (ii) Gleason grade group (Grade Group 1 [Gleason score 6] vs. Grade Group 5 [Gleason score 9–10]; n = 3 per group), and (iii) biochemical recurrence-free survival (long-term vs. short-term recurrence-free survival; n = 3 per group). These groupings were used to compare miRNA expression profiles associated with favorable versus unfavorable prognostic features. Given the limited sample size, these subgroup analyses were designed for exploratory comparison rather than definitive statistical inference. There were no significant age differences between paired subgroups (*p* > 0.05), whereas prognostic parameters differed significantly by design (*p* < 0.05).

### 2.2. miRNA Extraction

Total RNA, including small RNAs, was extracted from FFPE tumor sections using the miRNeasy Mini Kit (Qiagen, Hilden, Germany) according to the manufacturer’s instructions. Following deparaffinization and tissue lysis, RNA was eluted in 20 μL of RNase-free water. RNA concentration and integrity were assessed using a NanoDrop spectrophotometer (Thermo Fisher Scientific, Waltham, MA, USA) and agarose gel electrophoresis.

### 2.3. Library Preparation and Sequencing

Small RNA sequencing libraries were generated from approximately 10 ng of total RNA per sample using the SMARTer smRNA-Seq Kit for Illumina (Takara Bio, Shiga, Japan). Adapter ligation, reverse transcription with polyadenylation, and PCR amplification were performed according to the manufacturer’s protocol. Libraries were size-selected to enrich for fragments corresponding to mature miRNAs and assessed for quality and size distribution using an Agilent 2100 Bioanalyzer (Agilent Technologies, Santa Clara, CA, USA). Quantification was performed by qPCR, and equimolar pooled libraries were sequenced on an Illumina HiSeq 2500 platform (Illumina, San Diego, CA, USA) to generate single-end 101 bp reads. Sequencing and initial data processing were conducted by Macrogen (Seoul, Republic of Korea). The resulting data are available in the Gene Expression Omnibus (GEO) under accession number GSE179961.

### 2.4. miRNA Expression and Differential Analysis

Sequencing reads were quality-filtered and adapter-trimmed using Cutadapt (version 1.18) [18], and aligned to the human reference genome (GRCh38) and miRBase v21 using the miRDeep2 pipeline with Bowtie (version 1.2.3) [19]. miRNA expression levels were quantified as read counts per miRNA. Normalization was performed using the relative log expression (RLE) method in edgeR [20]. miRNAs with zero counts in more than 50% of samples were excluded. Differential expression analysis between prognostic groups was conducted using DESeq2 (version 1.36.0) [21], applying a fold-change threshold ≥2 and FDR < 0.05. Given the small sample size, this analysis was performed for exploratory candidate discovery, and the results should be interpreted with caution. The results were visualized using volcano plots and hierarchical clustering heatmaps. 

### 2.5. Target Prediction and Pathway Analysis

Predicted target genes of differentially expressed miRNAs were identified using TargetScan [22,23]. Functional enrichment analyses of KEGG pathways and Disease Ontology terms were performed using DAVID v6.8 [24], with *p* < 0.05 considered significant. Enrichment results were interpreted in the context of prostate cancer-related biological pathways. It should be noted that miRNA–mRNA relationships identified in this study are based on computational prediction algorithms and do not represent experimentally validated regulatory interactions.

Public transcriptomic datasets from TCGA-PRAD and GEO (GSE46602) were analyzed to evaluate expression patterns of selected target genes [25,26,27]. Patients were stratified into high- and low-risk groups based on Gleason score or biochemical recurrence status. Differential gene expression was assessed using Welch’s t-test with Benjamini–Hochberg correction. Data normalization, batch correction, and statistical analyses were performed in R (v4.2.1).

### 2.6. Statistical Analysis

Clinical variables were compared using Chi-square or Fisher’s exact tests for categorical data and Student’s *t*-test, ANOVA, or appropriate nonparametric tests for continuous variables. Results are presented as mean ± standard error, with *p* < 0.05 considered statistically significant. Due to the limited sample size, statistical analyses were interpreted in an exploratory context, and findings should be considered hypothesis-generating rather than confirmatory. Statistical analyses were conducted using R (version 4.2.1; R Foundation for Statistical Computing, Vienna, Austria) and SPSS software (version 26.0; IBM, Armonk, NY, USA).

## 3. Results

### 3.1. Patient Characteristics

Eighteen patients with prostate cancer were assigned to prognostic subgroups as described above, including low versus high risk, low versus high Gleason grade, and long versus short biochemical recurrence-free survival (n = 3 per subgroup). Clinical characteristics are summarized in Table 1. Patient age did not differ significantly between favorable and unfavorable prognosis groups in any comparison (all *p* > 0.05). In contrast, prognostic parameters differed significantly by design. The high-risk group had a significantly higher risk score than the low-risk group (2 ± 0 vs. 0 ± 0, *p* < 0.05). Similarly, patients with high-grade tumors had Gleason Grade Group 5 (Gleason score 9–10), whereas those in the low-grade group had Grade Group 1 (Gleason score 6; *p* < 0.05). Patients with short biochemical recurrence-free survival relapsed at a mean of 23.0 ± 8.4 days, compared with 4869.0 ± 84.9 days (~13 years) in the long recurrence-free group (*p* < 0.05), confirming the more aggressive disease characteristics of the unfavorable prognosis groups. Given the small sample size, these comparisons were designed to illustrate subgroup differences rather than provide definitive statistical conclusions.

### 3.2. Differential miRNA Expression by Risk Stratification

**Figure 1:**
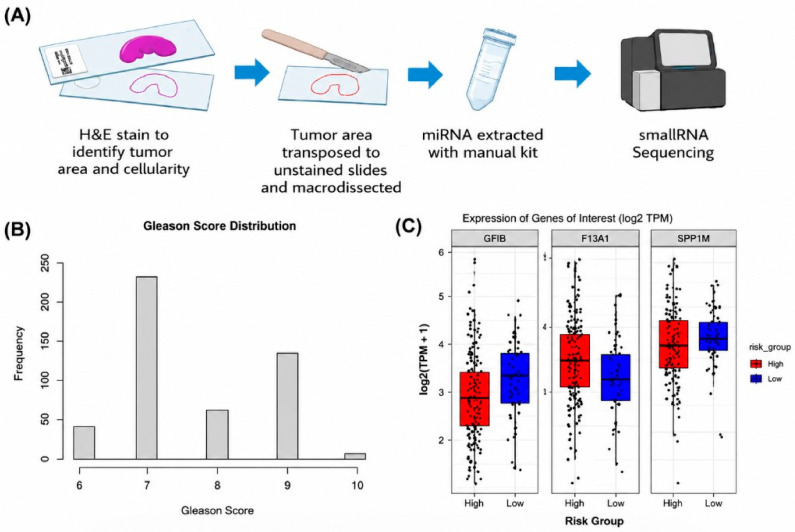
Study design and patient stratification workflow. Schematic overview of the study design. Prostate cancer FFPE tissue samples were stratified into prognostic groups based on (**A**) clinical risk category, (**B**) Gleason grade, and (**C**) biochemical recurrence-free survival. miRNA profiling was performed using NGS. This diagram illustrates the overall exploratory study design used for subgroup comparison.

**Figure 2:**
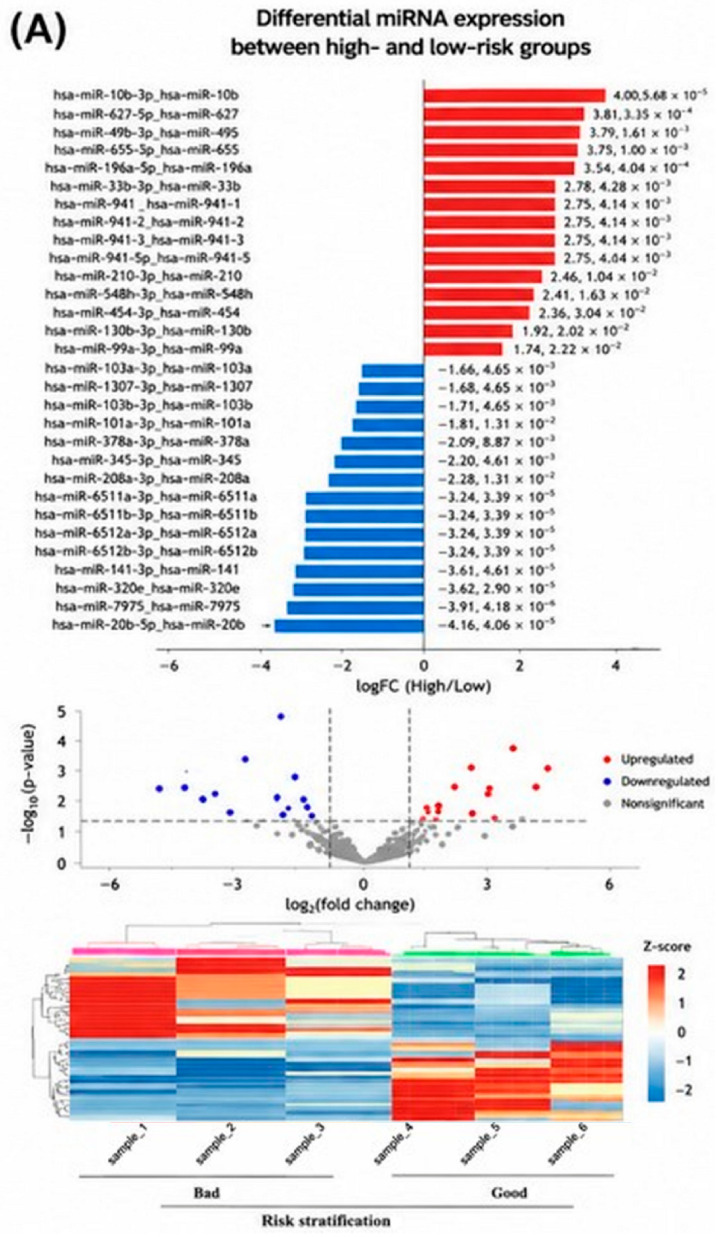

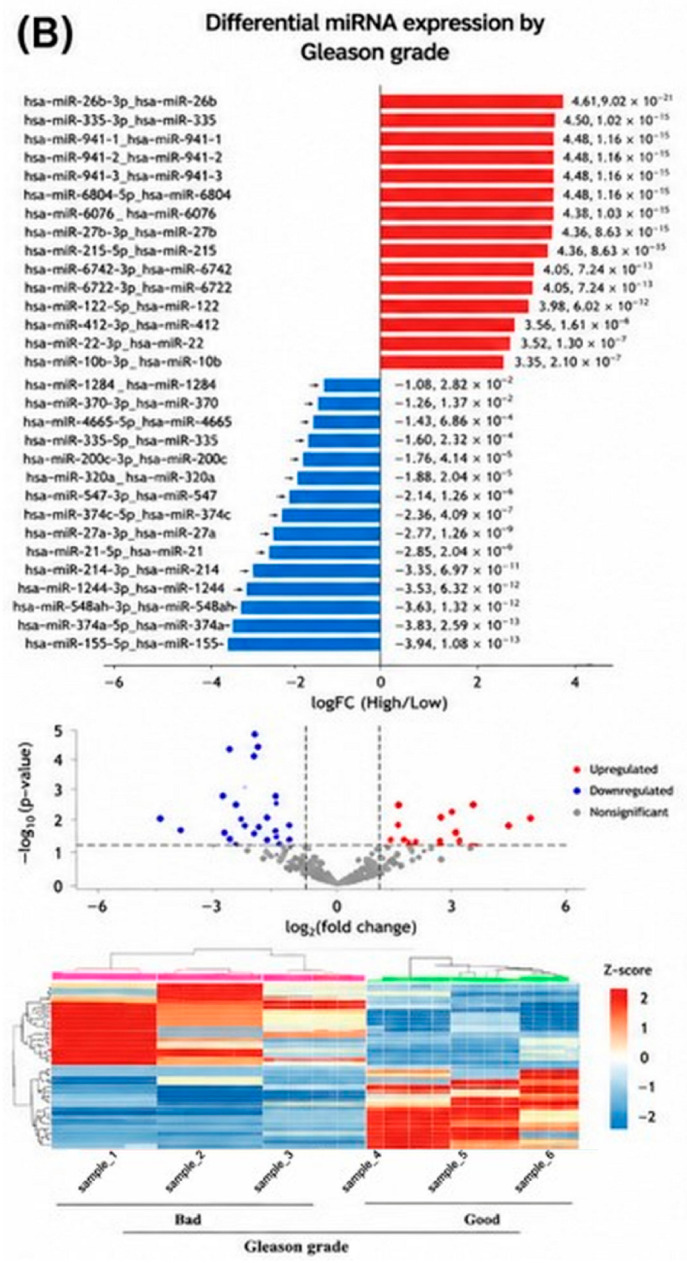

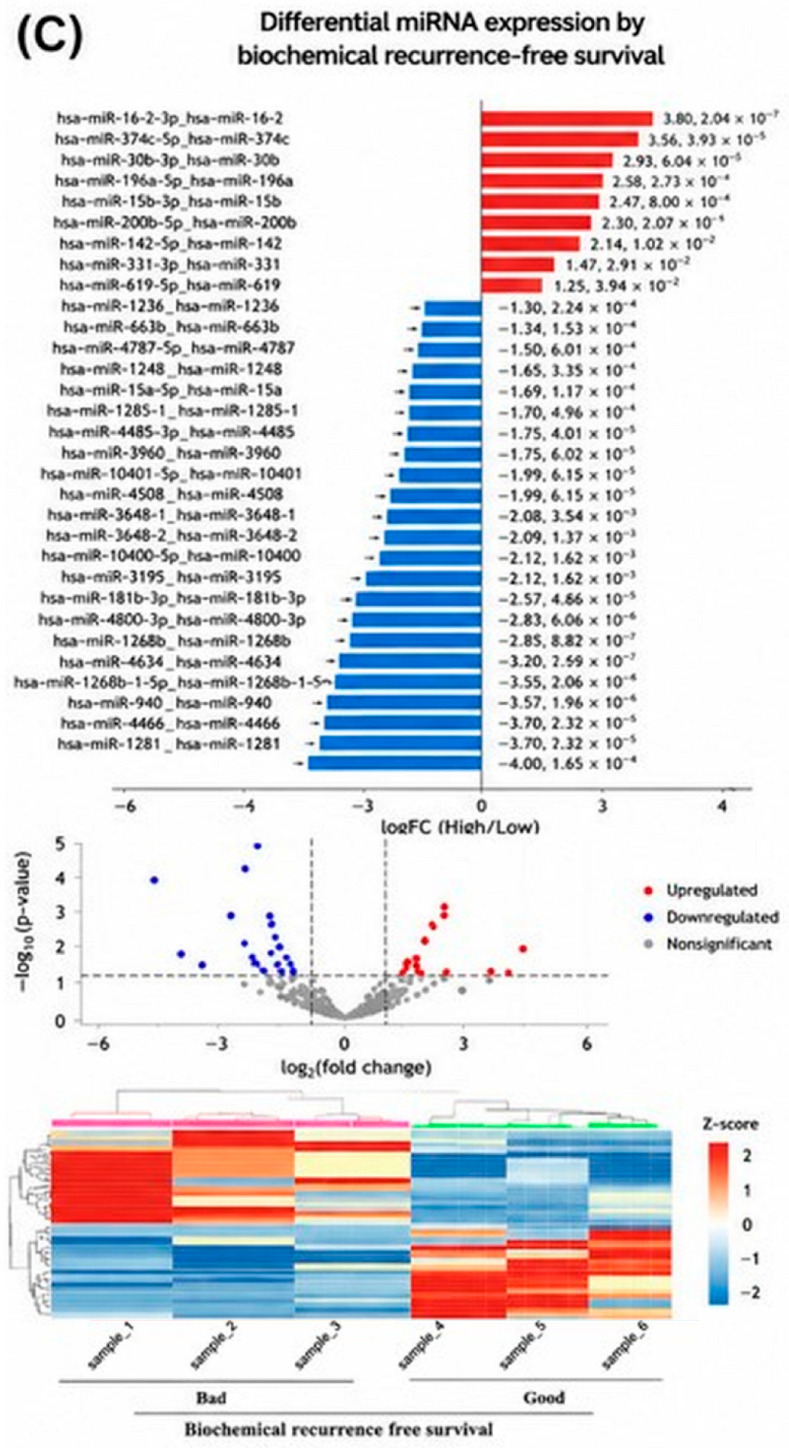
Differentially expressed miRNAs in prostate cancer tissues organized by prognostic category, with volcano plots and unsupervised clustering. (**A**) Risk stratification comparison (high-risk vs. low-risk patients). (**B**) Gleason grade comparison (high-grade vs. low-grade tumors). (**C**) Biochemical recurrence-free survival comparison (short vs. long relapse-free interval). In each volcano plot, red dots indicate miRNAs with *p* < 0.05 and fold-change ≥2 between the groups. The accompanying heatmaps show hierarchical clustering of samples based on expression of miRNAs that met the differential expression criteria (*p* < 0.05), illustrating distinct miRNA expression patterns between good and bad prognosis groups.

**Figure 3:**
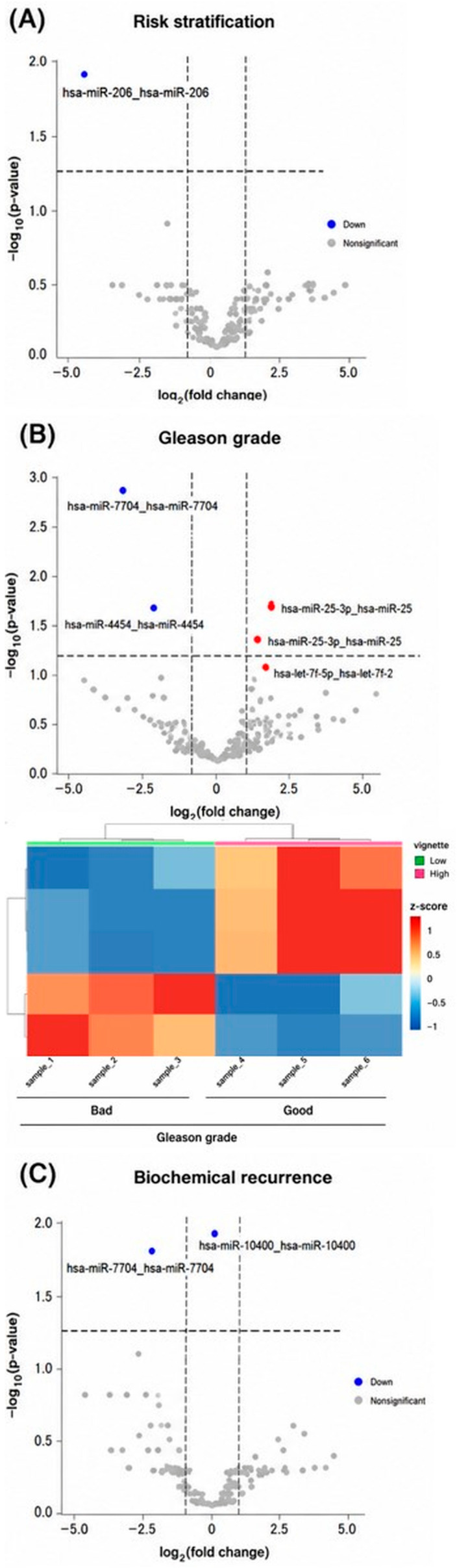
Key significant miRNAs associated with prognosis (fold-change ≥ 2 and FDR < 0.05). (**A**) Risk stratification: miR-206 was significantly down-regulated in high-risk versus low-risk prostate cancer (highlighted in the volcano plot and heatmap). (**B**) Gleason grade: five miRNAs (miR-7704, miR-4454, miR-25-3p, let-7f-5p from two loci) were significantly different between high-grade and low-grade tumors; miR-7704 and miR-4454 were down-regulated in high-grade tumors, whereas miR-25-3p and let-7f-5p were up-regulated. (**C**) Biochemical recurrence outcome: miR-7704 and miR-10400-5p were down-regulated in tumors from patients with short recurrence-free survival. Volcano plots show the distribution of miRNA changes with genome-wide significance, and heatmaps illustrate the expression clustering of these top prognostic miRNAs across individual patient samples.

The overall study design and patient stratification workflow are summarized in Figure 1. Comparison of miRNA profiles between low- and high-risk tumors identified multiple candidate miRNAs with nominally significant differential expression (*p* < 0.05). After correction for multiple testing, miR-206 emerged as the most significant candidate. miR-206 expression was markedly reduced in high-risk tumors compared with low-risk tumors (fold-change > 2, FDR < 0.05), suggesting a potential association between miR-206 downregulation and aggressive disease. These findings suggest a potential association between miR-206 downregulation and aggressive disease; however, this observation should be interpreted cautiously and requires validation in larger cohorts. These results are illustrated in the volcano plot and heatmap shown in Figure 2A and Figure 3A.

### 3.3. Differential miRNA Expression by Gleason Grade

Analysis by Gleason grade identified 89 miRNAs with nominal differential expression between low- and high-grade tumors. Among these, miR-7704, miR-4454, miR-25-3p, and let-7f-5p showed the strongest and most significant associations. miR-7704 and miR-4454 were significantly downregulated in high-grade tumors, whereas miR-25-3p and let-7f-5p (derived from the let-7f-1 and let-7f-2 loci) were upregulated. All four miRNAs exhibited ≥2-fold expression differences with FDR < 0.05 (Figure 2B and Figure 3B), suggesting candidate miRNA expression patterns that may be associated with tumor aggressive prostate cancer.

### 3.4. Differential miRNA Expression by Biochemical Recurrence

Comparison of tumors from patients with long versus short biochemical recurrence-free survival revealed 35 differentially expressed miRNAs (*p* < 0.05). miR-7704 and miR-10400-5p were significantly downregulated in tumors from patients who experienced early recurrence (fold-change > 2, FDR < 0.05; Figure 2C and Figure 3C). The observed expression patterns suggest potential associations with early recurrence; however, causal relationships cannot be established from this analysis. The consistent downregulation of miR-7704 in both high-grade and early-recurrence tumors suggests its potential relevance as a candidate marker of aggressive disease, requiring further validation.

### 3.5. Pathway and Disease Enrichment Analysis

**Figure 4:**
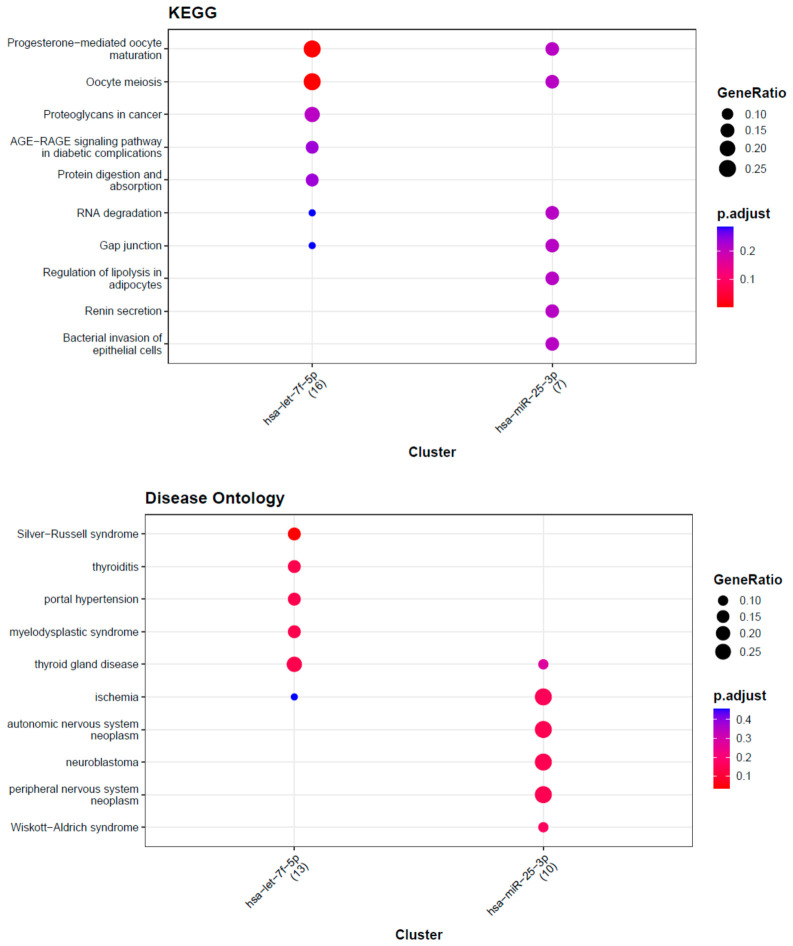
Functional enrichment analysis for the top five Gleason grade-associated miRNAs (miR-7704, miR-4454, miR-25-3p, let-7f-5p). Enriched KEGG pathways (upper) and Disease Ontology terms (lower) are shown for the predicted target genes of let-7f-5p (blue bars) and miR-25-3p (green bars). Notable pathways for let-7f-5p targets include progesterone-mediated oocyte maturation, oocyte meiosis, proteoglycans in cancer, AGE-RAGE signaling in diabetic complications, protein digestion/absorption, RNA degradation, and gap junction. For miR-25-3p targets, enriched pathways include RNA degradation, gap junction, regulation of lipolysis in adipocytes, renin secretion, and bacterial invasion of epithelial cells. Disease ontology enrichment indicates associations of let-7f-5p targets with developmental syndromes (Silver–Russell syndrome), endocrine and hematologic disorders (thyroiditis, myelodysplastic syndrome), etc., while miR-25-3p targets are associated with conditions like ischemia, certain neoplasms of neural tissues, and Wiskott–Aldrich syndrome. These analyses suggest that the dysregulated miRNAs may influence a variety of cancer-related pathways and disease processes.

Functional enrichment analysis of predicted targets of let-7f-5p and miR-25-3p revealed significant enrichment in KEGG pathways related to cancer-associated signaling, RNA degradation, gap junctions, metabolic regulation, and cell communication. Disease Ontology analysis linked let-7f-5p targets to developmental and immune-related disorders, while miR-25-3p targets were associated with diseases involving growth, neural development, and immune dysfunction. These findings suggest that the upregulated miRNAs may influence multiple pathways relevant to tumor progression (Figure 4).

### 3.6. miRNA–Target Interaction Network

**Figure 5:**
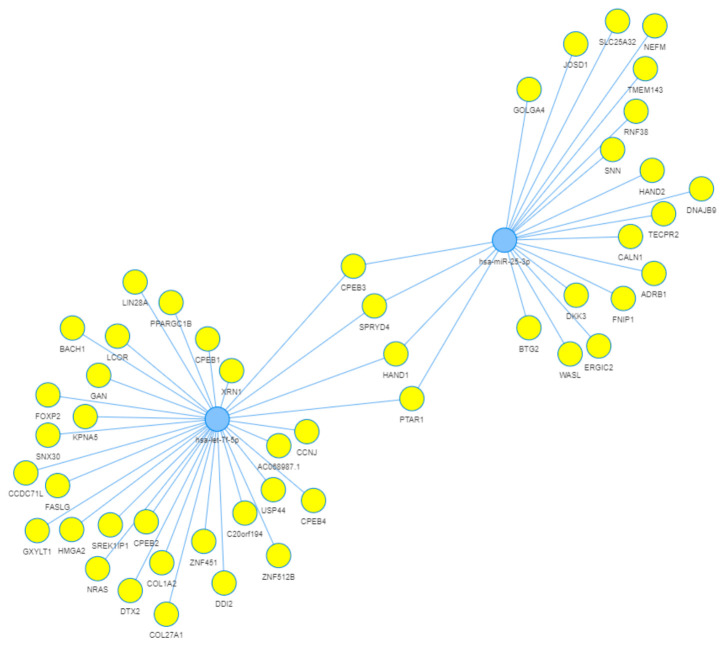
miRNA–gene interaction network for prognostic miRNAs in prostate cancer. The network focuses on the five miRNAs identified in the Gleason grade comparison. Blue nodes represent miRNAs (miR-7704, miR-4454, miR-25-3p, let-7f-5p), and yellow nodes represent their predicted target genes (based on TargetScan). Edges indicate a miRNA–mRNA targeting relationship. The network reveals that let-7f-5p and miR-25-3p regulate a broad set of genes, with some overlap. Four target genes—CPEB3, HAND1, PTAR1, and SPRYD4—are common targets of both let-7f-5p and miR-25-3p (highlighted in the center of the network). The network is based on prediction algorithms and does not represent experimentally validated regulatory relationships. Shared targets (e.g., CPEB3) are highlighted as candidate genes.

miRNA–mRNA network analysis demonstrated extensive regulatory interactions for let-7f-5p and miR-25-3p, including predicted targeting of cancer-related genes such as HMGA2, LIN28A, NRAS, DKK3, and BTG2. Four genes—CPEB3, HAND1, PTAR1, and SPRYD4—were identified as common targets of both miRNAs, indicating potential convergence points in miRNA-mediated regulation (Figure 5).

### 3.7. Validation in Public Cohorts

In the TCGA-PRAD cohort, CPEB3 was significantly downregulated in high-risk tumors (*p* = 0.00524, FDR = 0.00994), while PTAR1 was upregulated; SPRYD4 showed minimal change (Table 2). Consistently, analysis of the GSE46602 dataset confirmed significant downregulation of CPEB3 in high-risk patients (*p* = 0.00524, FDR = 0.00994), supporting its potential role as a prognostic marker (Table 3).

## 4. Discussion

In this study, we conducted comprehensive miRNA profiling of prostate cancer tissues and examined associations with established prognostic factors. In this exploratory analysis, our results suggest that prostate cancer aggressiveness may be associated with distinct miRNA expression patterns. Specifically, miR-206, miR-7704, miR-4454, miR-25-3p, and let-7f-5p were identified as candidate prognostic miRNAs, each showing significant differential expression between tumors with favorable and unfavorable clinical features. In addition, target prediction and network analyses highlighted several downstream genes, including SPRYD4 and CPEB3, as candidate mediators of miRNA-driven effects in aggressive prostate cancer.

One of the most notable findings was the marked downregulation of miR-206 in high-risk tumors. miR-206 has been reported to function as a tumor suppressor in prostate cancer and other malignancies. Prior work by Wang et al. demonstrated that miR-206 inhibits prostate cancer cell proliferation and migration by targeting CXCL11 and disrupting pro-tumorigenic signaling pathways [28]. Our observation of reduced miR-206 expression in aggressive tumors is consistent with this tumor-suppressive role, suggesting that loss of miR-206 may facilitate disease progression. However, given the exploratory design and limited sample size, this association should be interpreted cautiously and does not establish a causal relationship. Consequently, miR-206 may represent candidate biomarkers of aggressive prostate cancer, and CPEB3 is an RNA-binding protein involved in post-transcriptional regulation. High-Gleason-grade tumors displayed a more complex miRNA dysregulation pattern. miR-7704 and miR-4454 were significantly downregulated, whereas miR-25-3p and let-7f-5p were upregulated. Although miR-7704 is poorly characterized, its consistent downregulation in both high-grade and early-recurrence tumors suggests a potential tumor-suppressive function that is lost during disease progression. miR-4454 has been implicated as a tumor suppressor in other cancers. Studies in colorectal and ovarian cancer have shown that miR-4454 silencing promotes tumor progression, chemoresistance, and metastasis through pathways involving NF-κB signaling and metastatic niche interactions [29,30]. These findings extend prior observations in a hypothesis-generating manner and should not be interpreted as evidence of conserved biological function without further validation.

In contrast, miR-25-3p and let-7f-5p were upregulated in high-grade tumors. Although this appears counterintuitive given the generally tumor-suppressive roles attributed to these miRNAs, their functional effects are likely context-dependent. miR-25 has been reported to reduce invasiveness in aggressive prostate cancer cell lines by regulating integrin expression, thereby promoting a less metastatic phenotype [31]. Elevated miR-25-3p in high-grade tumors may therefore reflect compensatory responses, cellular stress, or cell-type-specific expression patterns within the tumor microenvironment. Further functional studies are needed to clarify whether miR-25 contributes to or counteracts tumor aggressiveness in prostate cancer.

Similarly, let-7f-5p, a member of the well-established let-7 tumor-suppressor family, was increased in high-grade tumors. Although let-7 family members are frequently downregulated in many cancers, prior studies have shown that let-7f can suppress proliferation, invasion, and metastasis in various contexts, including prostate and gastric cancers [32,33]. The increased expression observed in our cohort may reflect context-specific regulatory responses, tumor heterogeneity, or compensatory mechanisms rather than direct oncogenic or tumor-suppressive effects. Therefore, the functional significance of these findings remains uncertain and requires further investigation.

We also identified miR-7704 and miR-10400-5p as significantly downregulated in tumors from patients with early biochemical recurrence. Although both miRNAs are poorly characterized, their consistent underexpression in poor-outcome groups suggests that they may serve as candidate markers of aggressive disease, rather than definitive prognostic indicators. miR-10400-5p has been reported only sporadically in other disease contexts, and its function in cancer remains largely unexplored. Their biological roles remain largely unexplored, and further studies are needed to clarify their relevance in prostate cancer progression.

Pathway enrichment analysis of predicted miRNA targets suggested involvement in cell cycle regulation, intercellular communication, metabolic processes, and RNA stability. Targets of let-7f-5p were enriched in pathways related to cell division and junctional signaling, consistent with its known role in maintaining differentiated cell states. miR-25-3p targets were associated with metabolic regulation and signaling pathways, suggesting a role in tumor–microenvironment interactions. These findings are based on in silico prediction analyses and should be interpreted as hypothesis-generating rather than evidence of mechanistic pathways.

Network analysis identified four genes—CPEB3, HAND1, PTAR1, and SPRYD4—as common predicted targets of let-7f-5p and miR-25-3p. Among these, CPEB3 showed the most consistent association with aggressive disease, being significantly downregulated in high-risk tumors across both TCGA and GEO cohorts. CPEB3 is an RNA-binding protein involved in translational regulation and has been implicated as a tumor suppressor in other contexts [34]. HAND1 is a transcription factor involved in developmental regulation [35]. In the present study, however, CPEB3 should be interpreted as a prioritized candidate downstream target for future investigation, rather than a confirmed molecular target. SPRYD4 also emerged as a biologically compelling candidate. Post-translational modifications, including processes such as protein prenylation, play important roles in regulating protein localization and signaling functions in cancer-related pathways [36]. Accordingly, alterations in SPRYD4-associated regulatory mechanisms may contribute to tumor progression. Recent evidence suggests that SPRYD4 functions as a tumor suppressor in cholangiocarcinoma, where its loss is associated with poor prognosis and altered immune infiltration [37].

Our data indicate that SPRYD4 may be co-regulated by multiple prognostic miRNAs, raising the possibility that suppression of SPRYD4 contributes to aggressive prostate cancer behavior. Although changes in SPRYD4 expression were modest in public datasets, its predicted regulatory position warrants further functional validation. Importantly, recent studies have emphasized the clinical potential of miRNA-based biomarkers in prostate cancer; however, these studies also highlight the need for rigorous validation before clinical application [38].

Overall, our findings support the utility of miRNAs as prognostic biomarkers in prostate cancer. The distinct expression patterns of miR-206, miR-7704, miR-4454, miR-25-3p, and let-7f-5p across risk, grade, and recurrence suggest that miRNA-based signatures could complement existing clinical parameters to improve risk stratification. Moreover, identification of downstream targets such as CPEB3 provides insight into molecular pathways that may drive aggressive disease.

This study has several limitations. First, the extremely small sample size substantially limits statistical power and increases the risk of both false-positive and false-negative findings. Therefore, all results should be interpreted as preliminary and hypothesis-generating rather than definitive. Second, no orthogonal validation (e.g., qRT-PCR) was performed, and third, no functional experiments were conducted to establish causal relationships. These limitations are particularly important given that the present study primarily relies on computational analyses, which should be interpreted with caution. In line with previous reports emphasizing biomarker validation in prostate cancer, findings derived from small exploratory cohorts should be interpreted with caution until supported by large-scale and functionally validated studies [39]. Validation in larger, independent cohorts and prospective studies will be necessary to confirm the clinical relevance of these miRNAs. In particular, incorporation of minimal experimental validation approaches, such as qRT-PCR or independent expression correlation analysis, would substantially strengthen the robustness and biological credibility of the findings. Additionally, our analyses are correlative; functional experiments will be required to establish causal roles for these miRNAs and their targets in prostate cancer progression.

## 5. Conclusions

This exploratory NGS-based analysis of FFPE prostate cancer tissues identified candidate miRNA expression patterns that may be associated with prognostic features. Several miRNAs, including miR-206, miR-7704, miR-4454, miR-25-3p, and let-7f-5p, were associated with risk stratification, tumor grade, and biochemical recurrence. Target prediction and integrative analyses highlighted CPEB3, HAND1, PTAR1, and SPRYD4 as prioritized candidate downstream targets; however, these findings are based on computational and correlative analyses and do not establish causal relationships. Importantly, the extremely small sample size and the absence of orthogonal validation (e.g., qRT-PCR) and functional experiments substantially limit the interpretability of the results. Therefore, all findings should be regarded as preliminary and hypothesis-generating rather than definitive. Future studies incorporating larger independent cohorts and experimental validation will be essential to confirm the biological relevance and clinical applicability of these candidate miRNAs and their predicted targets.

## Figures and Tables

**Table 1:** Clinical characteristics of patients included in the miRNA differential expression analysis.

	Good (n = 3)	Bad (n = 3)	*p* Value
Risk stratification	0 ± 0	2 ± 0	<0.05
Age	62.3 ± 5.2	68.0 ± 3.1	>0.05
Gleason grade	1 ± 0	5 ± 0	<0.05
Age	68.3 ± 0.7	68.3 ± 0.7	>0.05
Biochemical recurrence-free survival	4869.0 ± 84.9	23.0 ± 8.4	<0.05
Age	65.3 ± 0.9	66.7 ± 4.8	>0.05

*p* < 0.05 indicates a statistically significant difference between good vs. bad prognosis groups for that factor (risk score, Gleason grade, or recurrence-free survival).

**Table 2:** Gene expression comparison in TCGA-PRAD (high-risk vs. low-risk prostate cancer).

Gene	Low-Risk Mean Expression	High-Risk Mean Expression	*p*-Value	FDR
*CPEB3*	3.4	3.1	0.00524 *	0.00994 *
*PTAR1*	3.2	3.5	0.00662 *	0.00994 *
*SPRYD4*	3.2	3.0	0.08041	0.08041

* *p* < 0.05, * FDR < 0.05 indicate a statistically significant difference between good vs. bad prognosis groups.

**Table 3:** Gene expression comparison in GSE46602 (high-risk vs. low-risk prostate cancer).

Gene	Low-Risk Mean Expression	High-Risk Mean Expression	*p*-Value	FDR
*CPEB3*	7.200738	6.560153	0.01660545 *	0.04981636 *
*PTAR1*	8.742913	8.456075	0.21304798	0.213047980
*SPRYD4*	6.413232	6.776050	0.17575247	0.21304798

* *p* < 0.05, * FDR < 0.05 indicate a statistically significant difference between good vs. bad prognosis groups.

## Data Availability

The data are not publicly available due to ethical restrictions. The data are, however, available from the corresponding author upon reasonable request.

## Abbreviations

The following abbreviations are used in this manuscript:

BPH	Benign prostatic hyperplasia
VAPB	The protein vesicle-associated membrane protein-associated protein B (VAPB)
PSA	Prostate-specific antigen (PSA)
miRNAs	MicroRNAs (miRNAs)
NGS	Next-generation sequencing (NGS)
FFPE	Formalin-fixed paraffin-embedded (FFPE)
H&E	Hematoxylin–eosin (H&E)
PCa	Prostate cancer (PCa)
FDR	False discovery rate
